# Automatic segmentation of myocardium at risk from contrast enhanced SSFP CMR: validation against expert readers and SPECT

**DOI:** 10.1186/s12880-016-0124-1

**Published:** 2016-03-05

**Authors:** Jane Tufvesson, Marcus Carlsson, Anthony H. Aletras, Henrik Engblom, Jean-François Deux, Sasha Koul, Peder Sörensson, John Pernow, Dan Atar, David Erlinge, Håkan Arheden, Einar Heiberg

**Affiliations:** Department of Clinical Physiology, Skåne University Hospital in Lund, Lund University, Lund, Sweden; Department of Biomedical Engineering, Faculty of Engineering, Lund University, Lund, Sweden; Laboratory of Medical Informatics, School of Medicine, Aristotle University of Thessaloniki, Thessaloniki, Greece; Department of Cardiology, Henri Mondor Hospital, Creteil, France; Department of Cardiology, Lund University, Lund, Sweden; Department of Medicine, Karolinska Institutet, Karolinska University Hospital, Stockholm, Sweden; Department of Cardiology B, Oslo, University Hospital Ullevål and Faculty of Medicine, University of Oslo, Oslo, Norway

**Keywords:** Quantification, Myocardial salvage, Randomized clinical trials, Expectation maximization, A priori information, Surface coil correction

## Abstract

**Background:**

Efficacy of reperfusion therapy can be assessed as myocardial salvage index (MSI) by determining the size of myocardium at risk (MaR) and myocardial infarction (MI), (MSI = 1-MI/MaR). Cardiovascular magnetic resonance (CMR) can be used to assess MI by late gadolinium enhancement (LGE) and MaR by either T2-weighted imaging or contrast enhanced SSFP (CE-SSFP). Automatic segmentation algorithms have been developed and validated for MI by LGE as well as for MaR by T2-weighted imaging. There are, however, no algorithms available for CE-SSFP. Therefore, the aim of this study was to develop and validate automatic segmentation of MaR in CE-SSFP.

**Methods:**

The automatic algorithm applies surface coil intensity correction and classifies myocardial intensities by Expectation Maximization to define a MaR region based on *a priori* regional criteria, and infarct region from LGE. Automatic segmentation was validated against manual delineation by expert readers in 183 patients with reperfused acute MI from two multi-center randomized clinical trials (RCT) (CHILL-MI and MITOCARE) and against myocardial perfusion SPECT in an additional set (*n* = 16). Endocardial and epicardial borders were manually delineated at end-diastole and end-systole. Manual delineation of MaR was used as reference and inter-observer variability was assessed for both manual delineation and automatic segmentation of MaR in a subset of patients (*n* = 15). MaR was expressed as percent of left ventricular mass (%LVM) and analyzed by bias (mean ± standard deviation). Regional agreement was analyzed by Dice Similarity Coefficient (DSC) (mean ± standard deviation).

**Results:**

MaR assessed by manual and automatic segmentation were 36 ± 10 % and 37 ± 11 %LVM respectively with bias 1 ± 6 %LVM and regional agreement DSC 0.85 ± 0.08 (*n* = 183). MaR assessed by SPECT and CE-SSFP automatic segmentation were 27 ± 10 %LVM and 29 ± 7 %LVM respectively with bias 2 ± 7 %LVM. Inter-observer variability was 0 ± 3 %LVM for manual delineation and -1 ± 2 %LVM for automatic segmentation.

**Conclusions:**

Automatic segmentation of MaR in CE-SSFP was validated against manual delineation in multi-center, multi-vendor studies with low bias and high regional agreement. Bias and variability was similar to inter-observer variability of manual delineation and inter-observer variability was decreased by automatic segmentation. Thus, the proposed automatic segmentation can be used to reduce subjectivity in quantification of MaR in RCT.

**Clinical trial registration:**

NCT01379261.

NCT01374321.

**Electronic supplementary material:**

The online version of this article (doi:10.1186/s12880-016-0124-1) contains supplementary material, which is available to authorized users.

## Background

Myocardium at risk (MaR) is defined as the ischemic myocardium during coronary artery occlusion, at risk of infarction if the blood flow in the occluded artery is not restored in time. The myocardial infarction evolves during time to treatment and if blood flow is not restored in time the whole region of MaR becomes myocardial infarction (MI). If both the size of MaR and final MI size is determined, the efficacy of reperfusion therapy can be assessed as myocardial salvage index (MSI = 1-MI/MaR). By using MSI instead of MI size alone the number of patients needed in clinical trials can be reduced [1] since MI size is related to MaR which is specific for each patient and coronary occlusion.

Cardiovascular magnetic resonance (CMR) is considered gold standard for assessment of infarct size by late gadolinium enhancement (LGE) [2]. Myocardial perfusion SPECT is considered gold standard for assessment of MaR but requires an radioactive isotope to be injected before the blood a flow is restored in occluded artery and imaging is performed only hours after the treatment. By CMR MaR can be assessed by either T2-weighted imaging [3] or contrast enhanced steady state free precession (CE-SSFP) [4] and both have been validated against SPECT for assessment of MaR up to one week after MI [4, 5]. Recently, both T2-weighted imaging and CE-SSFP have been used to determine myocardial salvage in two multi-center cardioprotective studies, CHILL-MI [6] and MITOCARE [7]. In these multi-center trials CE-SSFP was shown to provide significantly better diagnostic image quality than T2-weighted images and to be more robust across vendors [8]. CE-SSFP may therefore be more suitable than T2-weighted imaging for quantification of MaR in multi-center settings.

An automatic segmentation algorithm is preferable for objective quantification in order to reduce subjectivity as well as time required for image analysis. Several algorithms have been developed and validated for automatic segmentation of MI size in LGE images [3, 9, 10]. Two automatic algorithms have been developed and validated in T2-weighted images, one specifically for MaR [11] and one for edema [12]. However, no algorithm has been developed yet for quantification of MaR in CE-SSFP images. Automatic quantification of MaR in T2-weighted images has been shown to yield more accurate results when utilizing Expectation Maximization (EM) to classify myocardial intensities and adding an *a priori* model of the perfusion territories compared to thresholding methods such as two standard deviations (2SD) from remote, full width half maximum (FWHM) and Otsu’s method for quantification of MaR in T2-weighted images [11]. Therefore, the aim of this study was to develop and validate this automatic segmentation algorithm for MaR in CE-SSFP.

## Methods

### Study population and design

For validation of the automatic algorithm, patients with first time ST-elevation myocardial infarction (STEMI) treated with percutaneous coronary intervention (PCI) who had undergone CMR examination with CE-SSFP and LGE images of diagnostic quality as a part of the recently published clinical cardioprotection trials CHILL-MI [6] (*n* = 92) and MITOCARE [7] (*n* = 91) were included (*n* = 183). Patients underwent CMR imaging within 2-6 days following acute MI treated with PCI. Inclusion and exclusion criteria for each of the clinical trials have been previously published [6, 13]. In short, all patients had clinical signs of acute myocardial infarction defined as clinical symptoms and ECG signs consistent with ST-elevation infarction or new onset of left bundle branch block (LBBB), were ≥ 18 years old and had symptom duration of less than 6 h. Patients with a history of previous myocardial infarction or history of coronary revascularization were excluded.

For validation against an independent reference method of imaging MaR, an additional set of patients who had undergone both CE-SSFP CMR and single photon emission computed tomography (SPECT) (*n* = 16) [4] were included in this study. Inclusion and exclusion criteria for this cohort have also been previously published [4]. In short, all patients had clinical signs of acute myocardial infarction defined as clinical symptoms and ECG signs consistent with ST-elevation infarction and chest pain ≥ 30 min and ≤ 9 h. Patients with a history of previous myocardial infarction or history of coronary revascularization were excluded.

### Imaging

All CMR examinations were performed on 1.5 T scanners from Philips (Philips Healthcare, Best, The Netherlands), Siemens (Siemens AG, Erlangen, Germany) or GE (GE Healthcare, Waukesha, WI, USA). For visualization of MaR and evaluation of left ventricular function, CE-SSFP cine images were obtained approximately 5 min after intravenous injection of 0.2 mmol per kilogram of body weight of an extracellular gadolinium-based contrast agent [4, 6, 13]. The slice thickness was 8 mm with no slice gap. In-plane resolution was typically 1.5 x 1.5 mm. Typically, 20-30 CE-SSFP images were acquired per cardiac cycle. For infarct visualization LGE images covering the entire left ventricle were acquired approximately 15 min after injection of the gadolinium-based contrast agent. The LGE-images were acquired using an inversion-recovery gradient-recalled echo sequence with a slice thickness of 8 mm with no slice gap [14]. In-plane resolution was typically 1.5 x 1.5 mm. Inversion time was manually adjusted to null the signal of viable myocardium. Surface coil intensity correction was not mandatory across vendors and sites.

SPECT was performed in the additional set of 16 patients. Prior to opening the occluded vessel an intravenous injection of ^99m^Tc-tetrofosmin body weight adjusted (350-700 MBq) was administered to the patient. Myocardial perfusion SPECT imaging was performed within four hours to visualize and quantify MaR using either of two dual head cameras: GE (Ventri, GE Healthcare, Waukesha, WI, USA) or Sopha (DST-XL, Sopha Medical Vision, Bue, Cedex, France). Typical pixel size was 6.4 x 6.4 x6.4 mm (GE) and 3 x 3 x 3 mm (Sopha). Short axis images were reconstructed semi-automatically on the workstation for each camera.

### Image analysis

Both CMR and SPECT images were analyzed using the software Segment (http://segment.heiberg.se) [15].

In CE-SSFP images, MaR was manually assessed from short-axis images according to previously described methods [4, 6, 7]. In short, the left ventricular myocardium was defined by manually delineating the epicardial and endocardial borders both at end-diastole and at end-systole as previously described. Hyper-intense regions within the myocardium in CE-SSFP images were manually delineated for assessment of MaR. Hypo-intense myocardium within the area of increased signal intensity was regarded as microvascular obstruction [16] and was included in the MaR. The delineation of each data set was performed by one of three primary observers with a quality control of the delineations by a second opinion for each case. Different opinions for the delineation were resolved in consensus between all three observers when necessary. All three observers had long experience in the field of CMR (HE, MC and HA with 14, 15 and 20 years of experience, respectively). MaR was expressed as percent of left ventricular mass (%LVM) [17]. In a subset of 15 patients from the multi-center studies, second observer analysis was performed to evaluate inter-observer variability (MC vs. HE).

In LGE images, infarct was delineated from the short-axis images according to a previously validated method [9]. In short, the endocardial and epicardial borders were traced manually with exclusion of the papillary muscles. The LGE myocardium was defined using a previously validated automatic segmentation algorithm [9] which is based on a 1.8SD from remote threshold, region analysis and a weighted summation according to pixel intensities to take partial volume effects into consideration. Manual adjustments were made when obvious image artefacts caused misinterpretation by the automatic algorithm and to include micro vascular obstruction when not detected by the algorithm. Hypointense regions within the region of LGE as a sign of microvascular obstruction [16], were included in the analysis as 100 % infarction.

In SPECT images, MaR was delineated by use of an 55 % threshold [18] and manual corrections after automatic delineation of epicardial and endocardial borders [19]. MaR was expressed as percent of left ventricular mass (%LVM).

Image quality was manually assessed as (1) non-diagnostic, (2) acceptable or (3) good. Acceptable and good images were considered to be of diagnostic quality and only CE-SSFP images with diagnostic quality and full coverage of the left ventricle were included in this study as test set (*n* = 183, Additional file 1: Figure S1) and additional set (*n* = 16). Patient characteristics of the test set and additional set are reported in Table 1.

**Table 1:** Patient characteristics from test set *n* = 183

	Mean ± SD	(Min,max)
Heart rate [beats/min]	68 ± 12	(31, 111)
End diastolic volume [ml]	178 ± 43	(32, 336)
End systolic volume [ml]	94 ± 32	(20, 240)
Ejection fraction [%]	48 ± 9	(19, 70)
Left ventricular mass [g]	124 ± 28	(25, 252)
Infarct size [%LVM]	17 ± 10	(2, 47)
Microvascular obstruction [%LVM]	3 ± 5	(0, 27)

### Automatic segmentation algorithm

The automatic segmentation algorithm was originally developed for segmentation of MaR in T2-weighted images [11] and has in this study been developed for CE-SSFP images. Maximal extent models of perfusion territories for each coronary artery [11] were defined by expert observers and used to define remote and culprit region. The maximal extent models correspond to the MaR region of proximal occlusions and takes anatomy variations between patients into consideration. As input to the automatic algorithm, the manual delineation of endocardial and epicardial borders is used and the user defines the culprit artery as either left anterior descending artery (LAD), left circumflex artery (LCx), right coronary artery (RCA), or left main artery (LM) based on the overall appearance of the hyper enhanced region and defines right ventricular insertion points in CE-SSFP and LGE images, to find how to rotate the maximal extent model.

**Fig. 1:**
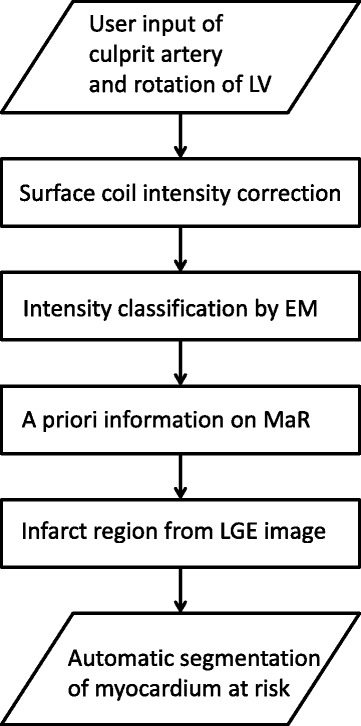
Automatic segmentation algorithm. The new automatic algorithm for segmentation of myocardium at risk (MaR) in CE-SSFP lets the user define the culprit artery and the rotation of the left ventricle as input. The algorithm consists of four processing blocks, surface coil intensity correction, intensity classification by Expectation Maximization (EM), segmentation based on a priori information on MaR and incorporation of infarct region from LGE images

The automatic algorithm consist of four processing blocks after user input as shown in Fig. 1, 1) surface coil intensity correction, 2) classification of myocardial intensities by Expectation Maximization (EM) [20], 3) definition of MaR region based on *a priori* regional criteria, and 4) incorporation of infarct region from LGE images. Surface coil intensity correction is applied as a second order linear correction based on the intensities in the blood pool and remote myocardium to be able to account for intensity gradient proportional to the squared coil distance. Classification of myocardial intensities is performed using the EM-algorithm to overcome varying contrast and noise level between patients, centers and vendors. The EM-algorithm estimates the mean and standard deviation of intensity for normal myocardium and myocardium at risk based on the intensity histogram and was initialized based on the maximal extent model. Myocardium at risk was defined as a continuous region within the maximal perfusion territory of the culprit artery and assumed to be transmural. These *a priori* regional criteria were implemented by applying the classification by EM sector wise for sectors within the maximal extent model. The myocardium is divided into 24 sectors circumferentially. Further *a priori* information was implemented by using the infarct region from LGE images to define possible regions of microvascular obstruction as MaR despite the hypoenhancement. The original algorithm for T2-weighted images [11] was based on intensity classification by Expectation Maximization (EM) and utilization of a priori information on MaR. Surface coil intensity correction and incorporation of the infarct region from LGE images was added in the new algorithm based on qualitative assessment of the CE-SSFP images. The new segmentation algorithm was named “Segment MaR CE-SSFP” and was implemented in the cardiac image analysis software Segment [9]. The algorithm will be made freely available at time of publication (http://segment.heiberg.se) and each processing block of the algorithm is further described in the Appendix.

### Comparison to other automatic threshold methods

The new automatic segmentation method was compared to three direct threshold methods which have been used for quantification of MaR in T2-weighted imaging [21, 22], two standard deviations from remote (2SD) [23, 24], full width half maximum intensity (FWHM) [25] and Otsu [26]. All methods used the same manual delineation of endocardium and epicardium. The 2SD threshold method estimates an intensity threshold from a remote region as the mean plus two standard deviations of the intensity within the remote region. The remote region was defined as the region outside the maximal extent model of the culprit artery [11]. The FWHM threshold method [27] estimates an intensity threshold from a remote region as midway between the mean intensity within the remote region and the maximal intensity within the myocardium. The remote region was defined in the same way as for 2SD. The threshold method of Otsu [28] estimates the intensity threshold from the histogram of all intensities to get minimal variance both above and below the threshold. For all three methods the intensity threshold was calculated and applied slice by slice as generally applied in T2-weighted images to account for the intensity gradient across slices.

### Statistical analysis

In the test set (*n* = 183) quantification of MaR by the automatic Segment MaR CE-SSFP algorithm was compared to the manual delineation using Bland-Altman bias (mean ± standard deviation), limits of agreement ([mean - 1.96 standard deviations; mean + 1.96 standard deviations]), and linear regression analysis (correlation coefficient). Regional agreement to manual delineation was evaluated by calculating Dice similarity coefficient (DSC) [29] (mean ± standard deviation). Dice similarity coefficient can be derived from the kappa statistics for classification of pixels [30] and is calculated as two times the volume of the intersection of the MaR regions divided by the sum of the volumes of the MaR regions. The DSC is therefore 0 if the regions do not overlap and 1 if the regions overlap perfectly. Bias, linear regression and regional agreement was similarly analyzed for the three automatic threshold methods, 2SD, FWHM and Otsu. Bias to manual delineation was analyzed separately for each of the three camera vendors for the automatic algorithm.

In a subset of 15 patients from the multicenter studies, inter-observer analysis of manual delineation and automatic segmentation was performed. Inter-observer analysis was assessed using Bland-Altman bias (mean ± standard deviation), linear regression (correlation coefficient) and regional agreement DSC (mean ± standard deviation) for manual delineation and automatic segmentation. Bias, linear regression and regional agreement was also assessed for automatic segmentation against manual delineation in the subset for comparison to inter-observer variability.

In the additional set (*n* = 16), quantification of MaR in CE-SSFP images by the automatic Segment MaR CE-SSFP algorithm and manual delineation was compared to quantification of MaR in SPECT using bias (mean ± standard deviation) and linear regression analysis (correlation coefficient).

The added value of each of the four processing blocks in the automatic algorithm described above was analyzed using bias (mean ± standard deviation), linear regression analysis (correlation coefficient), regional agreement DSC (mean ± standard deviation) and visualized by box-whisker plot of median, upper quartile, lower quartile, minimum, maximum and outliers. Two sided paired t-test of bias and DSC were performed for each processing block in comparison to the first block and the previous block with Bonferroni correction.

## Results

**Fig. 2:**
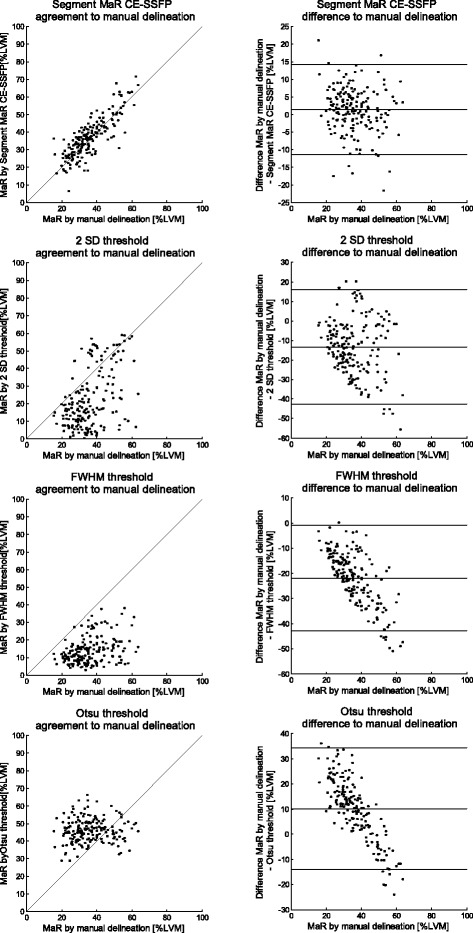
Correlation and bias for automatic segmentation and threshold methods against manual delienation. Correlation of MaR as % of LVM (left column) and Bland-Altman plot of MaR bias as % of LVM (right column) for the automatic segmentation algorithm (first row), threshold of 2SD from remote (second row), FWHM (third row) and Otsu (fourth row), all against manual delineation. The line of identity is shown as a solid line for all correlations plots and mean bias (solid line) and mean ± two standard deviations (dashed line) is shown for all Bland-Altman plots

**Fig. 3:**
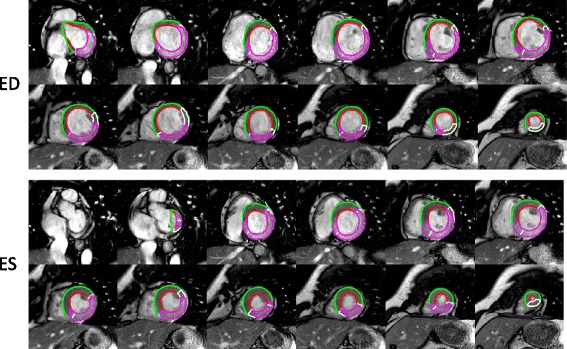
Example of automatic segmentation and manual delineation of MaR in CE-SSFP. Typical MaR segmentation in all left ventricular short axis slice images from one patient in end-diastole (ED, top panel) and end systole(ES, bottom panel), for automatic segmentation by Segment MaR CE-SSFP, shown in white, and manual delineation, shown in purple. Endocardial borders are shown in red and epicardial border in green. For this patient MaR by manual segmentation was 44 %LVM and by automatic Segment MaR CE-SSFP 43 % LVM with a regional agreement DSC of 0.85

In the test set (*n* = 183) MaR assessed by manual delineation in CE-SSFP was 36 ± 10 % LVM and MaR assessed by Segment MaR CE-SSFP automatic segmentation was 37 ± 11 %LVM. Bias was 1 ± 6 %LVM [-11; 14] %LVM, R = 0.83 and regional agreement DSC 0.85 ± 0.08 when Segment MaR CE-SSFP was compared to manual delineation (Fig. 2, Table 2). Figure 3 shows MaR in CE-SSFP at end-distole and end-systole with manual delineation and automatic segmentation by Segment MaR CE-SSFP. The bias was lower, regression stronger and regional agreement higher for Segment MaR CE-SSFP than for the threshold methods of 2SD, FWHM and Otsu (Fig. 2, Table 2). Bias to manual delineation analyzed per scanner vendors was 0 ± 7 %LVM, 2 ± 6 %LVM, and 2 ± 7 %LVM, for automatic segmentation in images from GE (*n* = 23), Philips (*n* = 76), and Siemens (*n* = 84), respectively. Inter-observer variability for manual delineation in CE-SSFP (*n* = 15) was 0 ± 3 %LVM compared to a bias between manual delineation and Segment MaR CE-SSFP of 2 ± 6 %LVM and inter-observer variability of Segment MaR CE-SSFP of -1 ± 2 %LVM (Table 3).

**Table 2:** Results from test set *n* = 183 for automatic Segment MaR CE-SSFP segmentation and threshold methods against manual delineation

	MaR bias [% of LVM]	Regression	DSC
		*R*-value	
Segment MaR CE-SSFP	1 ± 6	0.83	0.85 ± 0.08
2SD threshold	-13 ± 15	0.47	0.54 ± 0.27
FWHM threshold	-22 ± 11	0.42	0.42 ± 0.21
Otsu threshold	10 ± 12	0.05	0.65 ± 0.12

*MaR* Myocardium at risk, *LVM* Left ventricular mass, *DSC* Dice similarity coefficient, *Segment MaR CE-SSFP* automatic segmentation proposed in this study, *2SD* two standard deviations from remote, *FWHM* full width half maximum intensity

**Table 3:** Inter-observer variability analysis from subset *n* = 15 for manual delienation and automatic Segment MaR CE-SSFP segmentation compared to results for Segment MaR CE-SSFP against manual delineation

	MaR bias [% of LVM]	Regression	DSC
		*R*-value	
Manual delineation vs. manual delineation	0 ± 3	0.93	0.92 ± 0.04
Segment MaR CE-SSFP vs. Segment MaR CE-SSFP	-1 ± 2	0.99	0.94 ± 0.03
Segment MaR CE-SSFP vs. manual delineation	2 ± 6	0.77	0.86 ± 0.05

*MaR* Myocardium at risk, *LVM* Left ventricular mass, *DSC* Dice similarity coefficient, *Segment MaR CE-SSFP* automatic segmentation proposed in this study, manual delineation performed by a reference and a second observer, automatic Segment MaR CE-SSFP performed by a reference and a second observer

**Fig. 4:**
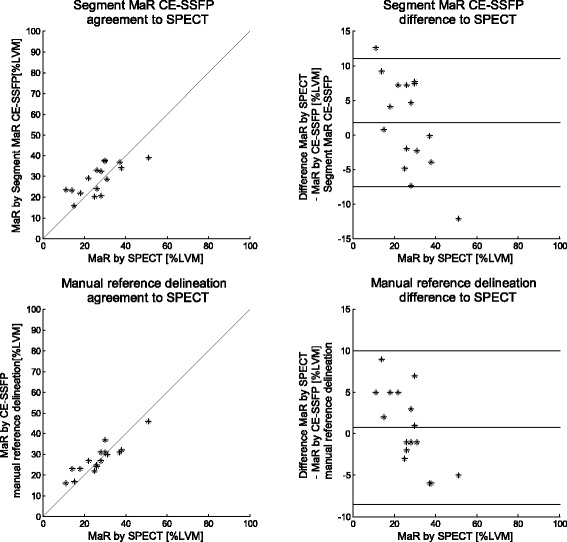
Correlation and bias against SPECT for automatic segmentation and manual delineation in CE-SSFP. Correlation of MaR as % of LVM (left column) and Bland-Altman plot of MaR bias as % of LVM (right column) against SPECT for automatic segmentation algorithm Segment MaR CE-SSFP (top row) and manual reference delineation (bottom row). The line of identity is shown as a solid line for all correlations plots and mean bias (solid line) and mean ± two standard deviations (dashed line) is shown for all Bland-Altman plots. Correlation and Bland-Altman plots for manual delineation in CE-SSFP against SPECT (bottom row) are adopted from Sorenson et al. [4]

In the additional set of patients (*n* = 16), MaR assessed by SPECT was 27 ± 10 %LVM. In CE-SSFP MaR was by manual delineation 28 ± 7 %LVM and by Segment MaR CE-SSFP 29 ± 7 %LVM. Bias against SPECT was 1 ± 5 %LVM (R = 0.90) for CE-SSFP by manual reference delineation and 2 ± 7 %LVM (R = 0.73) by Segment MaR CE-SSFP (Fig. 4).

**Fig. 5:**
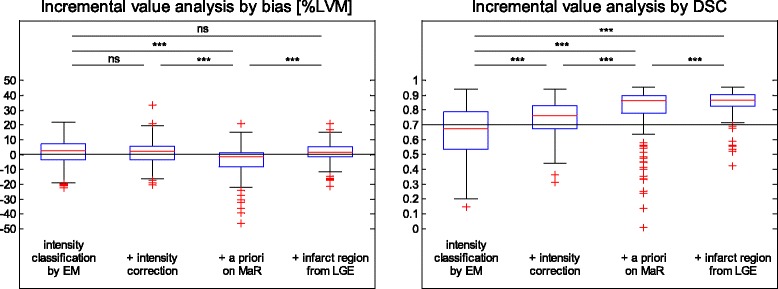
Analysis of incremental value of blocks in the automatic segmentation algorithm. Incremental value of each block in the automatic segmentation algorithm analyzed by bias to manual delineation as %LVM, left panel and by regional agreement as Dice similarity coefficient DSC (right panel). Bias and DSC was calculated with segmentation based on only intensity classification by Expectation Maximization and calculated after the addition of the processing blocks of intensity correction, a priori on myocardium at risk (MaR) and infarct region from late gadolinium enhancement (LGE). For each block of the algorithm the upper limit of the box indicate upper quartile, middle line indicate median, lower limit of box indicate lower quartile, whiskers indicate minimum and maximum points within 1.5 interquartile range and points (+) indicate outliers. Bias zero is shown as dotted black line in the left panel, DSC above of 0.7 indicates good regional agreement [30], and is shown as dotted black line in the right panel. Two sided paired t-test was performed for each block in comparison to previous block and first block, ns: non significant, ***: *p* < 0.0001

A significant difference in regional agreement DSC was shown for each of the processing blocks of the Segment MaR CE-SSFP algorithm even though the difference in bias %LVM was not significant (Fig. 5, Table 4).

**Table 4:** Analysis of incremental value of each block in the automatic *Segment MaR CE-SSFP* algorithm (*n* = 183)

	MaR bias [% of LVM]	Regression	DSC
		*R*-value	
Intensity classification by EM	2 ± 8	0.60	0.65 ± 0.18
+ intensity correction^a^	2 ± 8	0.63	0.74 ± 0.12
+ a priori on MaR	-4 ± 10	0.62	0.81 ± 0.16
+ infarct region from LGE	1 ± 6	0.83	0.85 ± 0.08

*EM* Expectation Maximization, *MaR* Myocardium at risk, *LGE* late gadolinium enhancement, *LVM* Left ventricular mass, *DSC* Dice similarity coefficient, ^a^applied in 127/183 patients

## Discussion

This study has presented an automatic algorithm for quantification of MaR in CE-SSFP images, validated against manual delineation in 183 patients from two multi-center, multi-vendor studies and against SPECT, as reference method, in 16 patients. The proposed automatic segmentation, Segment MaR CE-SSFP, shows low bias and variability, strong correlation and high regional agreement compared to manual delineation and SPECT. The Segment MaR CE-SSFP algorithm was shown superior to thresholding methods (2SD, FWHM and Otsu).

### Technical aspects

The added value of each processing block was shown significant by regional agreement DSC even though a significant difference in bias was only seen when bias changed from overestimation to underestimation adding use of *a priori* information on MaR. The significant change seen for DSC highlights the importance of analyzing regional agreement as a part of the validation in addition to bias.

Expectation Maximization was shown superior to 2SD, FWHM and Otsu, when considering regional agreement DSC, quantitative bias and correlation R-value. The EM-algorithm was used by Johnstone et al. [12] to find the mean and standard deviation of remote myocardium in T2-weighted black blood images, but the mean and standard deviation of edema was not used to define the threshold which may explain the lower regional agreement with DSC 0.50 ± 0.27. Gao et al. [31] also used the EM-algorithm to classify myocardial intensities in T2-weighted bright blood images, with the assumption of Rayleigh-Gaussian mixture model. Rayleigh distributed intensities were assumed due to nulling of remote myocardium [31] which is not done in CE-SSFP and therefore, in this study, Gaussian intensity distributions were assumed for both normal myocardium and MaR. Surface coil intensity correction was shown to increase regional agreement. The surface coil correction was based on intensities in remote myocardium and blood pool and thereby the bright blood property of CE-SSFP was advantageous to the black blood T2-STIR images in the original Segment MaR algorithm [11] where no intensity correction was applied. Surface coil correction was applied by the Segment MaR CE-SSFP algorithm if it resulted in reduced intensity variability in the remote myocardium and mean intensity in the culprit region higher than in the remote region. Surface coil correction was not mandatorily applied at the scanner and surface coil correction was applied by the Segment MaR CE-SSFP algorithm in a majority of the patients indicating that surface coil correction was either not applied at the scanner or not sufficient. Gao et al. [31] used intensity correction developed for the bright blood ACUT2E [32] images with use of proton density maps and achieved a DSC 0.7 ± 0.06 before applying feature analysis. By incorporating *a priori* regional criteria in the definition of the MaR region the regional agreement was further increased from 0.74 to 0.81. This is in line with Gao et al. [31] who showed increased regional agreement by DSC from 0.7 to 0.74 by adding feature analysis of the edema region. Both regional agreement by DSC and quantitative bias as %LVM was improved by the addition of information on the infarct region from LGE images which has not been implemented in previous studies. As for all automatic segmentation methods visual assessment and possibly manual corrections are needed and will probably influence the outliers seen after the fourth block of the algorithm and decrease the variability further.

### Comparison to previous studies

Regional agreement to manual delineation was for Segment MaR CE-SSFP higher than for the automatic segmentation methods by Johnstone et al. [12] (DSC 0.50 ± 0.27) and Gao et al. [31] (DSC 0.74 ± 0.06), and higher respectively similar to inter-observer regional agreement of manual delineation in the same studies (DSC 0.72 ± 0.14 [12] and 0.85 ± 0.03 [31]). Regional agreement of interobserver variability bias of Segment MaR CE-SSFP was comparable to inter-observer variability of manual delineation found in this study and similar to inter-observer variability previously found in CE-SSFP (2 ± 4 %LVM [4] and 0 ± 6 %LVM [17]), and in T2-weighted imaging (-2 ± 5 %LVM [11] and 5 ± 5 %LVM [17]). Bias of Segment MaR CE-SSFP to SPECT was low and comparable to the results from the validation study of CE-SSFP with manual delineation (0 ± 5 %LVM [4]). Bias was lower and regional agreement was higher for Segment MaR CE-SSFP than for the threshold methods of 2SD, FWHM and Otsu.

Recently McAlindon et al. [21] showed that manual delineation in T2-weighted images was superior to simple threshold methods (2, 3 and 5 SD from remote, FWHM and Otsu) with manual corrections regarding accuracy and variability of intra-observer, inter-observer and test-retest. Khan et al. [22] also showed that using simple threshold methods with manual corrections for inclusion of hypoenhancement and exclusion of artifacts did not significantly reduce time for analysis compared to manual delineation. Automatic segmentation methods are desirable to increase accuracy, decrease subjectivity and reduce time for analysis. Using simple threshold methods for MaR quantification in T2-weighted images seems to achieve neither and might be explained by the regional agreement seen in T2-weighted images (DSC 0.69 ± 0.14, 0.46 ± 0.14 and 0.68 ± 0.10 for 2SD, FWHM and Otsu respectively) [11] and in CE-SSFP images of this study the regional agreement for simple threshold methods was even lower. The regional agreement of the Segment MaR CE-SSFP was however similar to that of the original Segment MaR in T2-weighted images (DSC 0.85 ± 0.07) [11].

Segment MaR CE-SSFP was designed to include hypoenhancement and exclude artifacts and thereby has a greater potential to reduce time for analysis, and with a low bias to manual delineation by expert readers and a regional agreement and bias to manual delineation comparable to inter-observer of manual delineation Segment MaR CE-SSFP shows potential to increase accuracy and reduce subjectivity.

### Limitations

Limitations to the study are that test-retest scans were not performed and the effect of and time required for possible manual corrections following automatic segmentation was not evaluated. Contrast enhanced SSFP are not yet widely used for assessment of MaR but has been shown to be more robust than T2-weighted imaging in multi-center, multi-vendor studies [8] and can easily be implemented by acquiring cine SSFP images approximately 5 min after gadolinium injection.

## Conclusion

This study has presented an automatic algorithm, Segment MaR CE-SSFP for quantification of MaR in CE-SSFP images based on four processing blocks, Expectation Maximization, surface coil intensity correction, *a priori* regional criteria and incorporation of infarct region from LGE images. Low bias and variability, strong correlation and high regional agreement was shown against manual delineation in CE-SSFP images from multi-center, multi-vendor randomized clinical trials. Bias and variability was comparable to inter-observer variability of manual delineation and inter-observer variability was decreased by use of the Segment MaR CE-SSFP algorithm.

### Ethics approval and consent to participate

All three studies [4, 6, 7] from which patients were included were approved by the institutional review boards/ethics committees, and all patients provided written informed consent. No specific ethics approval or informed consent was needed for the development of the new automatic algorithm in the current study.

### Availability of data and materials

The new automatic algorithm is freely available for research purposes and can be downloaded from http://segment.heiberg.se.

## Figures and Tables

**Fig. 6:**
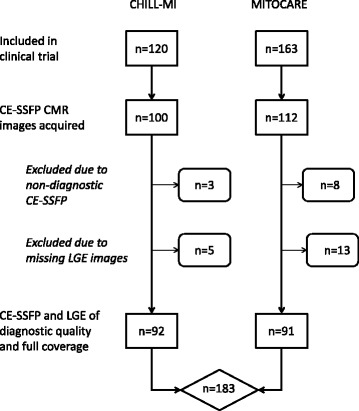
Model of maximal extent for perfusion territory of each culprit artery. Bulls-eye representation of maximal extent model for the perfusion territories of left anterior descending artery (LAD), left circumflex artery (LCx), right coronary artery (RCA), and left main artery (LM). Models for LAD, LCX and RCA were defined in consensus by three experienced observers in an extended 17- segment AHA model and models for LM were defined from the models of LAD, LCX and RCA. The 17-segment model is extended to three slices in each of the basal, mid-ventricular and apical zones and 24 sectors in each slice. Black sectors are included in the maximal extent model. The septal part of the left ventricle is represented in the left of the bulls-eye plot, the lateral part in the right, anterior part in the top, inferior part in the bottom, the apical slices in the center and the basal slices in the outer part of the bulls-eye plot

**Fig. 7:**
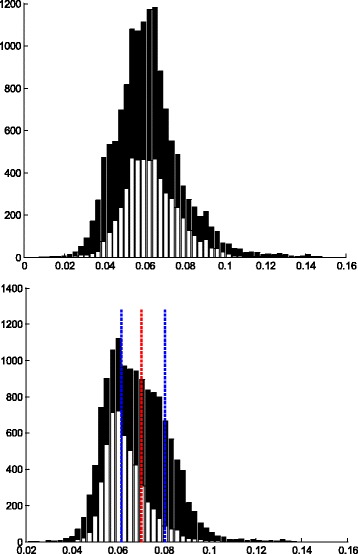
Surface coil intensity correction. Histogram of myocardial intensities within the myocardium before intensity correction (top panel) and after intensity correction (bottom panel), represented in black for culprit region and white for remote region. After intensity correction a decreased standard deviation of remote region is obtained and the mean intensity of the culprit region is higher than remote region. Myocardial intensities as after intensity correction (bottom panel) are used as input to the EM-algorithm with remote region and culprit region as initial classification and dashed blue lines indicating the constraints. Intensities below the 50th percentile of the remote region are kept classified as normal myocardium through the iterations of the EM-algorithm (lower dashed blue line). Intensities above the 75th percentile of the intensities within the culprit region are kept classified as MaR through the iterations of the EM-algorithm (upper dashed blue line). Dashed red line indicates the resulting cut off 0.5 of the Bayesian probability of MaR resulting from the EM-algorithm after intensity correction

**Fig. 8:**
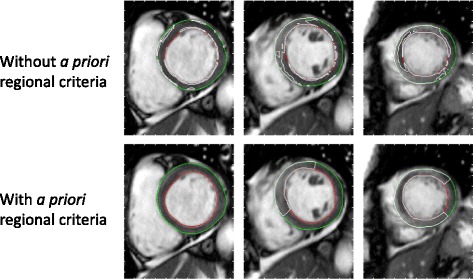
Utilization of *a priori* information on extent and transmurality. Three short axis slice, basal, mid and apical with MaR segmentation, shown in white, based on only the Bayesian probability of MaR (top row) and with the addition of *a priori* regional criteria on extent and transmurality (bottom row)

**Fig. 9:**
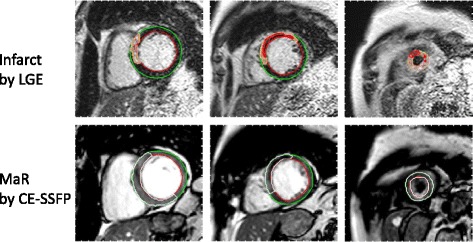
Utilization of infarct segmentation from LGE. Three short axis slices, basal, midventricular and apcial, from LGE (top row) with infarct region in yellow, infarct core in pink and microvascular obstruction in red, and CE-SSFP (bottom row) with automatic segmentation by Segment MaR CE-SSFP after utilization of information on infarct region from LGE. The large region of hypoenhancement in CE-SSFP is included as MaR by utilizing the segmentation of infarct region

## Appendix 1

### Detailed description of the automatic segmentation method

The automatic segmentation Segment MaR CE-SSFP was developed for segmentation of MaR in CE-SSFP based on ideas from the algorithm developed for T2-weighted images [12]. The use of Expectation Maximization (EM) algorithm [21] for classification of myocardial intensities [12] was improved with modified constraints and surface coil intensity correction. For definition of the MaR region the implementation of *a priori* regional criteria [12] was improved and utilization of information on infarct region from LGE images was added. Maximal extent models for the perfusion territory of the culprit artery [12] were used to define the remote region and the culprit region based on user input. Figure 6 shows the maximal extent models as defined in consensus by three experienced observers [12]. As input to the automatic algorithm, the user defines the culprit artery as either LAD, LCx, RCA or LM based on the overall appearance of the hyper enhanced region and defines right ventricular insertion points in CE-SSFP and LGE images, to define maximal extent model and how to rotate the model. The maximal extent model is used with the user input of culprit artery and LV rotation to define the remote myocardium for surface coil intensity correction and initialization of the EM-algorithm and to define a MaR region within the maximal perfusion territory.

Varying surface coil sensitivity may cause an intensity gradient through the CMR images and can in CE-SSFP cause a larger variability in the myocardium than the contrast between MaR and normal myocardium and hence a surface coil correction needs to be applied before the EM-algorithm. A second order intensity correction is applied to account for a gradient proportional to the squared distance to the surface coil. The correction is calculated based on the intensity in the remote myocardium and blood pool with papillaries excluded from the blood pool by using a simple unconstrained EM-algorithm. The intensity correction should result in a reduced intensity variability in the remote myocardium and a mean intensity in the culprit region higher than in the remote region, otherwise the correction is not applied. If the mean intensity in the remote myocardium is higher than in the culprit region both before and after the intensity correction, no correction is applied and the user is notified with a warning on low image quality. Figure 7 shows the intensity histogram before and after intensity correction for the remote and culprit region.

For classification of pixel intensities as normal myocardium or MaR, a Bayesian probability is calculated by the use of a constrained EM-algorithm. The EM-algorithm [21] iteratively refines an initial classification to find the maximum likelihood estimate of the mean and standard deviation for the intensity distributions of normal myocardium and MaR. The initial classification is defined from the maximal extent model with all pixels in the remote region initially classified as normal myocardium and all pixels in the culprit region initially classified as MaR. The EM-algorithm was constrained to keep the initial classification of normal myocardium for pixels with intensity below the 50th percentile in the remote region, respectively, keeping classification of MaR for pixels with intensity above the 75th percentile in the culprit region. The Bayesian MaR probability is calculated for each myocardial pixel as the intensity distribution of MaR divided by the sum of the intensity distributions of MaR and normal myocardium. The resulting Bayesian MaR probability cutoff 0.5 indicates higher probability of MaR and is shown in the histogram after intensity correction in Fig. 7.

The MaR region is defined as a connected region with high MaR probability which fullfills the *a priori* cirteria on transmurality and localization within the culprit artery’s perfusion territory. The mean MaR probability is calculated for each sector in a bullseye representation with 24 sectors and 30 interpolated slices averaged over the time frames. Sectors with a mean MaR probability above 0.5 which are within the maximal extent and connected to its nearest neighboring sector within the slice or in an adjacent slice in a 4-neighbourhood constitute a region. If several connected regions are found the region with highest summed MaR probability is chosen. Gray scale morphological operations of opening and closing are applied to remove holes and small peninsulas in a 4-neighbourhood. Additionally holes within slices are removed to account for larger encapsulated regions of microvascular obstruction. Non-physiological extent in apical and basal slices is detected for LAD and LM as missing apical MaR sectors or false basal sectors and for LCx and RCA as false apical sectors. False and missing sectors was detected as extent larger than mean + 2 standard deviations respectively smaller than mean - 2 standard deviations of the extent in midventricular slices. If any non-physiological extent was detected and corrected for, then the user is notified to check correctness of MaR region in the basal or apical slices. Figure 8 shows one short axis slice with MaR segmentation before and after applying *a priori* regional criteria.

If LGE images with infarct segmentation are available the information on the infarct region can be used as part of the *a priori* regional criteria for definition of the MaR region. The infarct region is always a part of MaR but may due to hypoenhancment of microvascular obstruction not always be detected as MaR by the EM-algorithm. From the LGE images with delineation of the infarct, either by manual delineation or automatic segmentation [9], and right ventricular insertion points the infarct region, represented as a sector-wise bullseye, is used to define the MaR region. For each sector the fraction of infarct is calculated and the MaR region is defined from sectors with either the infarct fraction above 0.5 or mean MaR probability above 0.5. Figure 9 shows a short axis slice of a CE-SSFP image with a distinct region of microvascular obstruction which can be determined as MaR region by the use of the infarct segmentation from the LGE images.

From the bulls eye representation of the MaR region a MaR segmentation is defined in the short axis slices for each time frame and MaR is expressed as %LVM averaged over end-diastole and end-systole.

## Additional file

Additional file 1: Figure S1. Patient inclusion from clinical trials. Patient inclusion from clinical trials CHILL-MI and MITOCARE resulted in 183 patients in the test set. In total 29 patients with CE-SSFP images were excluded due to non-diagnostic image quality or missing LGE images. (PDF 57 kb)

## Abbreviations

%LVM: Percent of left ventricular mass
CE-SSFP: Contrast enhanced SSFP
DSC: Dice similarity coefficient
EM: Expectation maximization
FWHM: Full width half maximum
LAD: Left anterior descending artery
LCx: Left circumflex artery
LGE: Late gadolinium enhancement
LM: Left main artery
MaR: Myocardium at risk
MI: Myocardial infarction
MSI: Myocardial salvage index
RCA: Right coronary artery
RCT: Randomized clinical trials
SPECT: Single photon emission computed tomography

## References

[1] Engblom H, et al. Sample size in clinical cardioprotection trials using myocardial salvage index, infarct size or biochemical markers as endpoint. J Am Heart Assoc. 2016. In Press. 10.1161/JAHA.115.002708 PMC4943247 26961520

[2] Kim RJ (1999). Relationship of MRI delayed contrast enhancement to irreversible injury, infarct age, and contractile function. Circulation.

[3] Aletras AH (2006). Retrospective determination of the area at risk for reperfused acute myocardial infarction with T2-weighted cardiac magnetic resonance imaging: histopathological and displacement encoding with stimulated echoes (DENSE) functional validations. Circulation.

[4] Sorensson P (2010). Assessment of myocardium at risk with contrast enhanced steady-state free precession cine cardiovascular magnetic resonance compared to single-photon emission computed tomography. J Cardiovasc Magn Reson.

[5] Carlsson M (2009). Myocardium at risk after acute infarction in humans on cardiac magnetic resonance: quantitative assessment during follow-up and validation with single-photon emission computed tomography. JACC Cardiovasc Imaging.

[6] Erlinge D (2014). Rapid endovascular catheter core cooling combined with cold saline as an adjunct to percutaneous coronary intervention for the treatment of acute myocardial infarction. The CHILL-MI trial: a randomized controlled study of the use of central venous catheter core cooling combined with cold saline as an adjunct to percutaneous coronary intervention for the treatment of acute myocardial infarction. J Am Coll Cardiol.

[7] Atar D (2015). Effect of intravenous TRO40303 as an adjunct to primary percutaneous coronary intervention for acute ST-elevation myocardial infarction: MITOCARE study results. Eur Heart J.

[8] Nordlund D, et al. Multi-vendor, multicentre comparison of contrast-enhanced SSFP and T2-STIR CMR for determining myocardium at risk in ST-elevation myocardial infarction*.* Eur Heart J Cardiovasc Imaging. 2016. doi:10.1093/ehjci/jew027. 10.1093/ehjci/jew027 PMC4907382 27002140

[9] Heiberg E (2008). Automated quantification of myocardial infarction from MR images by accounting for partial volume effects: animal, phantom, and human study. Radiology.

[10] Amado LC (2004). Accurate and objective infarct sizing by contrast-enhanced magnetic resonance imaging in a canine myocardial infarction model. J Am Coll Cardiol.

[11] Sjogren J (2012). Semi-automatic segmentation of myocardium at risk in T2-weighted cardiovascular magnetic resonance. J Cardiovasc Magn Reson.

[12] Johnstone R.I, et al. Assessment of tissue edema in patients with acute myocardial infarction by computer-assisted quantification of triple inversion recovery prepared MRI of the myocardium. Magn Reson Med. 2011;66(2):564-73. 10.1002/mrm.22812 21394767

[13] Atar D, Abitbol JL, Arheden H, Berdeaux A, Bonnet JL, Carlsson M, Clemmensen P, Cuvier V, Danchin N, Dubois-Randé JL, Engblom H, Erlinge D, Firat H, Eggert Jensen S, Halvorsen S, Hansen HS, Heiberg E, Larsen AI, Le Corvoisier P, Longlade P, Nordrehaug JE, Perez C, Pruss R, Sonou G, Schaller S, Tuseth V, Vicaut E. Rationale and design of the ‘MITOCARE’ Study: a phase II, multicenter, randomized, double-blind, placebo-controlled study to assess the safety and efficacy of TRO40303 for the reduction of reperfusion injury in patients undergoing percutaneous coronary intervention for acute myocardial infarction. Cardiology. 2012;123(4):201-7. 10.1159/000342981 23202613

[14] Simonetti OP (2001). An improved MR imaging technique for the visualization of myocardial infarction. Radiology.

[15] Heiberg E (2010). Design and validation of Segment--freely available software for cardiovascular image analysis. BMC Med Imaging.

[16] Beek AM, Nijveldt R, van Rossum AC (2010). Intramyocardial hemorrhage and microvascular obstruction after primary percutaneous coronary intervention. Int J Cardiovasc Imaging.

[17] Ubachs JF (2012). Myocardium at risk by magnetic resonance imaging: head-to-head comparison of T2-weighted imaging and contrast-enhanced steady-state free precession. Eur Heart J Cardiovasc Imaging.

[18] Ugander M (2012). Quantification of myocardium at risk in myocardial perfusion SPECT by co-registration and fusion with delayed contrast-enhanced magnetic resonance imaging--an experimental ex vivo study. Clin Physiol Funct Imaging.

[19] Soneson H (2009). An improved method for automatic segmentation of the left ventricle in myocardial perfusion SPECT. J Nucl Med.

[20] Dempster AP, Laird NM, Rubin DB (1977). Maximum likelihood from incomplete data via em algorithm. J R Stat Soc B-Methodol.

[21] McAlindon E, et al. Quantification of infarct size and myocardium at risk: evaluation of different techniques and its implications. Eur Heart J Cardiovasc Imaging. 2015;16(7):738-46. 10.1093/ehjci/jev001 PMC4463003 25736308

[22] Khan JN (2015). Comparison of semi-automated methods to quantify infarct size and area at risk by cardiovascular magnetic resonance imaging at 1.5T and 3.0T field strengths. BMC Res Notes.

[23] Friedrich MG (2008). The salvaged area at risk in reperfused acute myocardial infarction as visualized by cardiovascular magnetic resonance. J Am Coll Cardiol.

[24] Wright J (2009). Quantification of myocardial area at risk with T2-weighted CMR: comparison with contrast-enhanced CMR and coronary angiography. JACC Cardiovasc Imaging.

[25] Tilak GS (2008). In vivo T2-weighted magnetic resonance imaging can accurately determine the ischemic area at risk for 2-day-old nonreperfused myocardial infarction. Invest Radiol.

[26] Burchell T (2011). Comparing analysis methods for quantification of myocardial oedema in patients following reperfused ST-elevation MI. J Cardiovasc Magn Reson.

[27] Hsu LY (2006). Quantitative myocardial infarction on delayed enhancement MRI. Part I: Animal validation of an automated feature analysis and combined thresholding infarct sizing algorithm. J Magn Reson Imaging.

[28] Otsu N (1979). Threshold selection method from gray-level histograms. IEEE Trans Syst Man Cybern.

[29] Dice LR (1945). Measures of the amount of ecologic association between species. Ecology.

[30] Zijdenbos AP (1994). Morphometric Analysis of white-matter lesions in Mr-Images - method and validation. IEEE Trans Med Imaging.

[31] Gao H (2013). Highly automatic quantification of myocardial oedema in patients with acute myocardial infarction using bright blood T2-weighted CMR. J Cardiovasc Magn Reson.

[32] Aletras AH (2008). ACUT2E TSE-SSFP: a hybrid method for T2-weighted imaging of edema in the heart. Magn Reson Med.

