# Supplementary material for: Automatic segmentation of myocardium at risk from contrast enhanced SSFP CMR: validation against expert readers and SPECT
Source: BMC Med Imaging. 2016 Mar 5;16:19. doi: 10.1186/s12880-016-0124-1 (PMC4779553; doi:10.1186/s12880-016-0124-1)
Supplement: Additional file 1: Figure S1. — Patient inclusion from clinical trials. Patient inclusion from clinical trials CHILL-MI and MITOCARE resulted in 183 patients in the test set. In total 29 patients with CE-SSFP images were excluded due to non-diagnostic image quality or missing LGE images. (PDF 57 kb) [file 12880_2016_124_MOESM1_ESM.pdf]

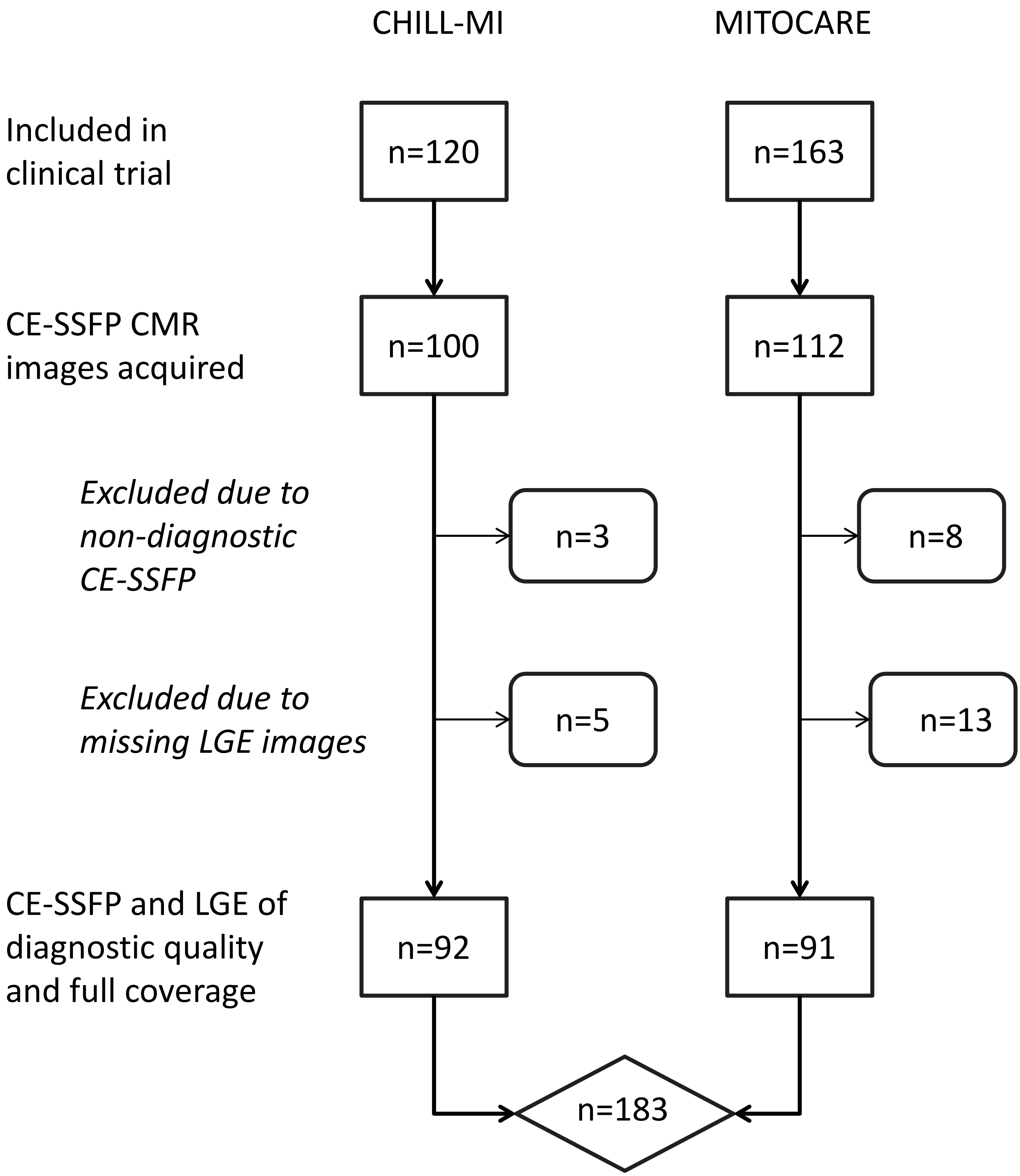
CHILL-MI
MITOCARE
Included in clinical trial
n=120
n=163
CE-SSFP CMR images acquired
n=100
n=112
Excluded due to non-diagnostic CE-SSFP
n=3
n=8
Excluded due to missing LGE images
n=5
n=13
CE-SSFP and LGE of diagnostic quality and full coverage
n=92
n=91
n=183
